# Electrospun Composites Made of Reduced Graphene Oxide and Polyacrylonitrile-Based Activated Carbon Nanofibers (rGO/ACNF) for Enhanced CO_2_ Adsorption

**DOI:** 10.3390/polym12092117

**Published:** 2020-09-17

**Authors:** Faten Ermala Che Othman, Norhaniza Yusof, Javier González-Benito, Xiaolei Fan, Ahmad Fauzi Ismail

**Affiliations:** 1 Advanced Membrane Technology Research Centre (AMTEC), School of Chemical and Energy Engineering, Faculty of Engineering, Universiti Teknologi Malaysia, Johor Bahru 81310, Malaysia; fermala2@live.utm.my (F.E.C.O.); norhaniza@petroleum.utm.my (N.Y.); afauzi@utm.my (A.F.I.); 2 Department of Materials Science and Engineering and Chemical Engineering, IQMAAB, Universidad Carlos III de Madrid, Avda. Universidad 15, Leganés, 28911 Madrid, Spain; 3 Department of Chemical Engineering and Analytical Science, School of Engineering, The University of Manchester, Manchester M13 9PL, UK; xiaolei.fan@manchester.ac.uk

**Keywords:** activated carbon nanofibers (ACNF), reduced graphene oxide (rGO), rGO/ACNF composite, CO_2_ capture, electrospinning

## Abstract

In this work, we report the preparation of polyacrylonitrile (PAN)-based activated carbon nanofibers composited with different concentrations of reduced graphene oxide (rGO/ACNF) (1%, 5%, and 10% relative to PAN weight) by a simple electrospinning method. The electrospun nanofibers (NFs) were carbonized and physically activated to obtain activated carbon nanofibers (ACNFs). Texture, surface and elemental properties of the pristine ACNFs and composites were characterized using various techniques. In comparison to pristine ACNF, the incorporation of rGO led to changes in surface and textural characteristics such as specific surface area (*S*_BET_), total pore volume (*V*_total_), and micropore volume (*V*_micro_) of 373 m^2^/g, 0.22 cm^3^/g, and 0.15 cm^3^/g, respectively, which is much higher than the pristine ACNFs (e.g., *S*_BET_ = 139 m^2^/g). The structural and morphological properties of the pristine ACNFs and their composites were studied by Raman spectroscopy and X-ray diffraction (XRD), and field emission scanning electron microscopy (FE-SEM) respectively. Carbon dioxide (CO_2_) adsorption on the pristine ACNFs and rGO/ACNF composites was evaluated at different pressures (5, 10, and 15 bars) based on static volumetric adsorption. At 15 bar, the composite with 10% of rGO (rGO/ACNF0.1) that had the highest *S*_BET_, *V*_total_, and *V*_micro_, as confirmed with BET model, exhibited the highest CO_2_ uptake of 58 mmol/g. These results point out that both surface and texture have a strong influence on the performance of CO_2_ adsorption. Interestingly, at *p* < 10 bar, the adsorption process of CO_2_ was found to be quite well fitted by pseudo-second order model (i.e., the chemisorption), whilst at 15 bar, physisorption prevailed, which was explained by the pseudo-first order model.

## 1. Introduction

The massive emissions of anthropogenic carbon dioxide (CO_2_) gas into the atmosphere are considered as the main reason for the occurrence of global warming and climate change [1,2]. Human activities such as the combustion of fossil fuels in industry, especially in the power generation sector, are major emission sources of CO_2_ into the atmosphere [3,4]. In mid-August 2020, according to the latest update from Mouna Loa Observatory [5], the increment of CO_2_ concentration was recorded as 3.02 ppm from August 2019 to August 2020 and reached up to 412.97 ppm as compared to the previous year’s concentration, i.e., 409.95 ppm, which is an alarming rate, since CO_2_ concentration in the atmosphere at <350 ppm is considered safe [6]. There was a temporary reduction in daily global CO_2_ emissions during the COVID-19 forced confinement in April 2020 reported by Le Quéré et al. (2020), but this does not really reflect structural changes in the economic, transport or energy system [7]. Various agreements have been developed among nations worldwide, including the Kyoto Protocol and Paris Agreement, to face the challenges caused by carbon emission, especially CO_2_ emission. Accordingly, this has encouraged many research efforts around the world to develop advanced materials, techniques and strategies to address the problems associated with CO_2_ emission. Popular strategies have been explored and adopted, including the utilization of low carbon fuels and renewable energy sources, and CO_2_ capture, storage and utilization from their source points [8].

Carbon capture and storage (CCS) is a promising approach to mitigate anthropogenic CO_2_ with the capacity to reduce up to 22% of CO_2_ emissions in 2035 [3]. These past few decades, absorption is the common method in CCS for CO_2_ capture and separation from flue gases in power plants. However, CO_2_ absorption via amine scrubbing possesses apparent disadvantages, such as the release of toxic gases and chemicals, high energy requirement for regeneration, and extensive corrosion of the equipment, which limit the practical application of this technology. Although amine-scrubbing has been widely employed in industrial applications, due to their disadvantages, the development of practical yet sustainable alternatives is still highly desired [9,10]. Consequently, other alternative and effective methods, such as adsorption, have been suggested, due to its simplicity in operation [2], low energy requirement, ease of regeneration, environmental-friendly, and cost-effective [11]. Adsorption employs solid porous adsorbents such as silica [12], zeolites [13], activated carbon [11], metal organic frameworks (MOF) [14,15,16], carbon nanotubes (CNT) [17], metal oxides [18], and graphene [19], to adsorb adsorbate molecules, especially CO_2_, onto the porous structures. Amongst the available adsorbents being investigated, porous ones such as activated carbons (ACs) were preferred due to their low cost, high surface area and porosity, high adsorption capability, high amenability to modify the pore structure and functionalize the surface, low energy requirements for regeneration, as well as hydrophobicity [20,21].

ACs in granular and powdered form are the most commonly used adsorbent [21]. Generally, ACs have relatively low micropore volume and multimodal pore size distribution, which are the main factors to limit their adsorption capabilities. Conversely, in comparison with the conventional ACs, newly developed fibrous ACs, also known as activated carbon nanofibers (ACNFs) [22], have shown the improved adsorption capacity due to the fibrous structure and presence of accessible micropores from their external surface [23], which reduce the mass transfer resistance for adsorbate diffusion to reach the adsorption sites. Polyacrylonitrile (PAN) is the most used polymer in electrospinning of NFs due to its high carbon yield and thermal-stable at high temperature treatments. Although the developed pristine PAN-based ACNFs has shown the improved adsorption performance as compared with the commercial ACs, recent study disclosed that the inclusion of nanofillers/additives could further improve the surface area and micropore volume of the modified ACNFs [24,25]. In comparison with other additives, graphene and graphene oxide with novel properties and economical carbon-based materials have been the potential candidates for adsorbent materials due to their large theoretical specific surface area (*S*_BET_) and high porosity [26,27]. These excellent properties have opened up the utilization of graphene in a wide range of applications, including supercapacitors, biomedicals, fuel cells, energy storage etc. [28]. Accordingly, graphene-based composites such as nitrogen-doped graphene oxide sheets (N-GOs) have been proposed by Alghamdi et al. (2018) [29] for energy storage applications related to gas adsorption and separation. They have observed that the N-GOs demonstrated a good CO_2_ adsorption capacity of 1.36 mmol/g. Notably, the selection of GO in this study is also because the GO can be mass-produced from inexpensive graphite as raw materials and a cost-effective method which made the reduced GO (rGO) composites into economical adsorbents. 

Here, this work reports the preparation of novel carbonaceous composites of reduced graphene oxide (rGO) doped PAN-based activated carbon nanofibers, i.e., rGO/ACNF using a simple electrospinning method and pyrolysis process. Up to now, there is no publication that extensively discussed the utilization of PAN-based rGO/ACNFs composites for CO_2_ adsorption. Therefore, the main novelty of this present work is to develop and characterize the rGO/ACNF composites (with different rGO loadings) using different techniques to understand their physicochemical properties and assess for CO_2_ adsorption (using a volumetric adsorption system at 5–15 bar) to evaluate their potentials for CCS. Kinetics of CO_2_ adsorption of the materials under investigation are also studied in detail to understand their adsorption mechanisms and performance.

## 2. Materials and Methods

### 2.1. Synthesis of Reduced Graphene Oxide (rGO)

The graphite powder (~325 mesh, 99%) was purchased from Alfa Aesar, and other chemicals such as sulphuric acid (H_2_SO_4_), sodium nitrate (NaNO_3_), potassium permanganate (KMnO_4_), hydrogen chloride (HCl), and hydrogen peroxide (H_2_O_2_) were obtained from Merck and used without further purification. Graphene oxide (GO) was prepared from natural graphite powder using a conventional Hummer’s method. Then, 150 mL of concentrated H_2_SO_4_ was added into the mixture of graphite powder and NaNO_3_. The suspension was vigorously stirred at <20 °C in an ice bath, followed by the addition of KMnO_4_ (18 g). Then, the temperature of the solution was increased slowly to 35 °C, and the mixture was stirred for another 30 min before it was quenched by adding 300 mL of deionized (DI) water. The temperature of the mixture was then raised to 98 °C and stirred overnight. Afterward, 300 mL of 30% H_2_O_2_ was introduced into the mixture, which was followed by the addition of 5% HCl solution to remove any metal ions left over [30]. The resulting solid was washed several times with DI water (until the neutral pH was achieved), separated by vacuum filtration and dried under vacuum (at 50 °C for 24 h) to obtain the GO. To obtain rGO, the GO sample was thermally reduced and exfoliated under thermal condition at >800 °C and inert atmosphere [31,32].

### 2.2. Preparation of Activated Carbon Nanofibers Nanocomposites (gACNFs)

rGO (1, 5 and 10 wt. %, respectively, relative to the weight of polymer polyacrylonitrile, PAN; 150,000 molecular weight of kDa) was dispersed in dimethylformamide (DMF; 99.999%) and sonicated under stirring for 24 h at room temperature until the homogenous solution was obtained. Both polymer and solvent were procured from Sigma-Aldrich. PAN (8 wt. % relative to the total weight) was added into the solution, and the solution was stirred again for 24 h at room temperature to obtain a homogenous solution [25]. Detailed formulation was shown in Table S1 (Supplementary Materials). 

PAN-based NFs were prepared using the optimum electrospinning conditions established by our previous research [30]. Specifically, the injection flow rate was 1.0 mL/h, voltage power was 10 kV, and the distance between the needle tip and collector was 15 cm. Furthermore, the ambient parameters inside the chamber including the humidity, temperature, and air velocity were also controlled during the operation. The obtained pristine and composite nanofibers (with 1, 5, and 10 wt. % rGO loading) were denoted as NF, rGO/NF0.01, rGO/NF0.05, and rGO/NF0.1, respectively. Activated carbon nanofibers (ACNF) were produced by subjecting the electrospun NFs to three stages of pyrolysis, including thermal stabilization (oxidation), carbonization, and activation, where the experimental details were described elsewhere [25,33]. The activated NFs were denoted as ACNF, rGO/ACNF0.01, rGO/ACNF0.05, and rGO/ACNF0.1 accordingly.

### 2.3. Characterization of Materials

Porous structures of the materials under investigation were characterized by using nitrogen (N_2_) adsorption/desorption analysis at −196 °C on Micromeritics Tristar II 3020, and the specific surface area (*S*_BET_) was calculated using the Brunauer–Emmett–Teller (BET) method. Prior N_2_ sorption analysis, the sample was degassed at 100 °C under vacuum for at least 24 h. The total pore volume (*V*_total_) was measured from the adsorption amounts of N_2_ gas at *p*/*p*_0_ = 0.99 and the micropore volume (*V*_micro_) was determined by density functional theory (DFT). The mesopore volumes (*V*_meso_) were calculated by subtracting the *V*_micro_ from *V*_total_. The structural transformation and degree of graphitization of the materials were analyzed using Raman spectroscopy (on Raman Xplora Plus spectrometer). To study the phase structure and crystallinity of the materials, an X-ray diffractometer (XRD, Rigaku SmartLab; Rigaku Corporation, The Woodlands, TX, United States) was used. Surface morphology and diameter, as well as the elemental composition of the materials, were analyzed using field electron scanning emission microscope coupled with elemental dispersive X-ray spectroscopy (FE-SEM-EDX, Hitachi SU8020; Hitachi High-Technologies Corporaation, Tokyo, Japan). Prior to FE-SEM analysis, all samples were dried at 100 °C and stored in a desiccator overnight.

### 2.4. CO_2_ Adsorption Experiments

**Figure 1:**
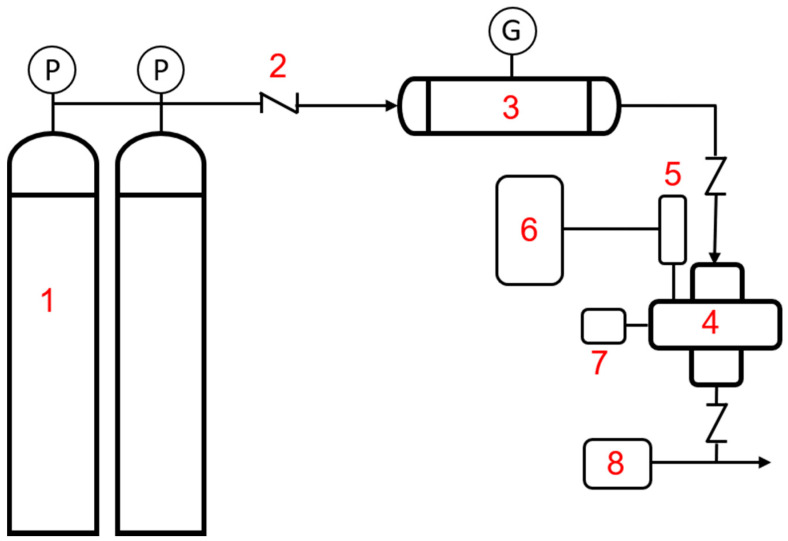
Schematic diagram of the volumetric set-up for CO_2_ adsorption. **1**. Feed gas **2**. Check valve **3**. Loading column **4**. Adsorption column **5**. High pressure transducer **6**. Pressure meter **7**. Thermocouple **8**. Vacuum pump; **P**: pressure regulator; **G**: pressure gauge.

Static volumetric CO_2_ adsorption measurements were performed on the same rig as the one shown in Figure 1. Prior to CO_2_ adsorption, the sample (~0.5 g) was dried under vacuum at 150 °C for 12 h to remove the moisture. In each test, the ACNFs and CO_2_ were loaded into adsorption cell (AC) and loading cell (LC), respectively, until the pressure achieved the required levels. The gas adsorption test was started once the pressure reached the desired level (5, 10 and 15 bar), by introducing the CO_2_ (adsorbates) with the ACNFs (adsorbent) in the AC by turning the valve between the AC and LC. The selection of these three pressures is to determine the adsorption capacity of both pristine and composite ACNFs at low and moderate pressures. The pressure and temperature in both AC and LC were recorded every 5 min until the pressure reached equilibrium. The adsorption equilibrium was achieved when both pressure and temperature were constant for approximately 10 min. The amount of CO_2_ adsorbed was calculated by using Equation (1):

(1) $$q=\;\frac{1}{m}\left[\frac{Vv}{R}{\left({\left|\frac{P}{ZT}\right|}_{i}-\;{\left|\frac{P}{ZT}\right|}_{eq}\right)}_{a}+\;{\left({\left|\frac{P}{ZT}\right|}_{i}-\;{\left|\frac{P}{ZT}\right|}_{eq}\right)}_{l}\right]$$

where *m* is the adsorbent mass (g), *q* is the amount of gas adsorbed (mmol/g), *P* is pressure (bar), *T* is temperature (K), *V* is volume (cm^3^), *R* is the gas constant, while the subscripts *a*, *l*, *i*, and *eq* refer to adsorption cell, loading cell, the initial state and adsorption final equilibrium state, respectively. *Z* is the compressibility factor [34].

The CO_2_ adsorption performance of pristine ACNF and composite were evaluated as a function of time, until equilibrium is reached at room temperature and different adsorption pressures of 5, 10, and 15 bar. In this study, only a rGO/ACNF0.1 composite was used for further assessment (kinetic studies) to compare with the pristine ACNFs, due its comparatively high N_2_ and CO_2_ adsorption performance and the associated high *S*_BET_ value.

### 2.5. Kinetic Studies

Apart from the study of adsorption capacity, when a new system is evaluated in terms of its potentiality for industrial applications, an understanding of adsorption kinetics is also necessary. In principle, kinetic parameters should provide information about mass transfer rates of the adsorbates trough the external adsorbed layer or layers, macropores and micropores of the adsorbent. Up to now, literature provides several kinetic models [11,35,36]. In general, experimental data obtained are fitted with the functions associated to those models to finally select the best fitting to choose the model describing the kinetics of the adsorption process.

Lagergren’s pseudo-first order and pseudo-second order of kinetic models were used to study the experimental data of CO_2_ adsorption on the materials at different times. The pseudo-first order assumes that the adsorption rate is proportional to the number of free adsorption sites on the surface of the adsorbent. The linear equation of this model is shown as follows:

(2) $$ln\left(q_{e}-\;q_{t}\right)=\;\ln q_{e}-k_{1}t$$

where *q*_t_ is the weight-specific adsorbed amount of the adsorbate at time *t* (s) during the adsorption process (mmol/g), *q*_e_ is the weight-specific adsorbed amount of the adsorbate at the end of equilibrium (mmol/g), and *k*_1_ is the adsorption rate constant of the pseudo-first order model.

Therefore, if the representation of $ln\left(q_{e}-\;q_{t}\right)$ versus time can be well fitted to a straight line, then it can be said that adsorption of CO_2_ obeys the pseudo-first order model. In general, this pseudo-first order model explains reversible interactions between adsorbent and adsorbate and therefore accounts for physisorption processes [11].

On the other hand, the pseudo-second order considers that the adsorption rate is proportional to the square of vacant adsorption sites on the adsorbent surface and the linear equation, as shown in Equation (3).

(3) $$\frac{t}{q_{t}}=\;\frac{1}{k_{2}q_{e}^{2}}\;+\frac{1}{q_{e}}t$$

where $k_{2}$ (mmol/g) is the adsorption rate constant for the pseudo-second order equation. Therefore, if the representation of *t*/*q*_t_ versus time can be well fitted to a straight line, then it can be said that adsorption of CO_2_ obeys the pseudo-second order model. This pseudo-second order model explains relatively strong interactions, chemical interactions, between the gas molecules and the adsorbent, therefore the process would be represented by a chemisorption as the rate controlling step [37]. Both $k_{1}$ and $k_{2}$ values can be obtained from the slopes of the corresponding fitted straight lines.

## 3. Results and Discussion

### 3.1. Properties of Materials

**Figure 2:**
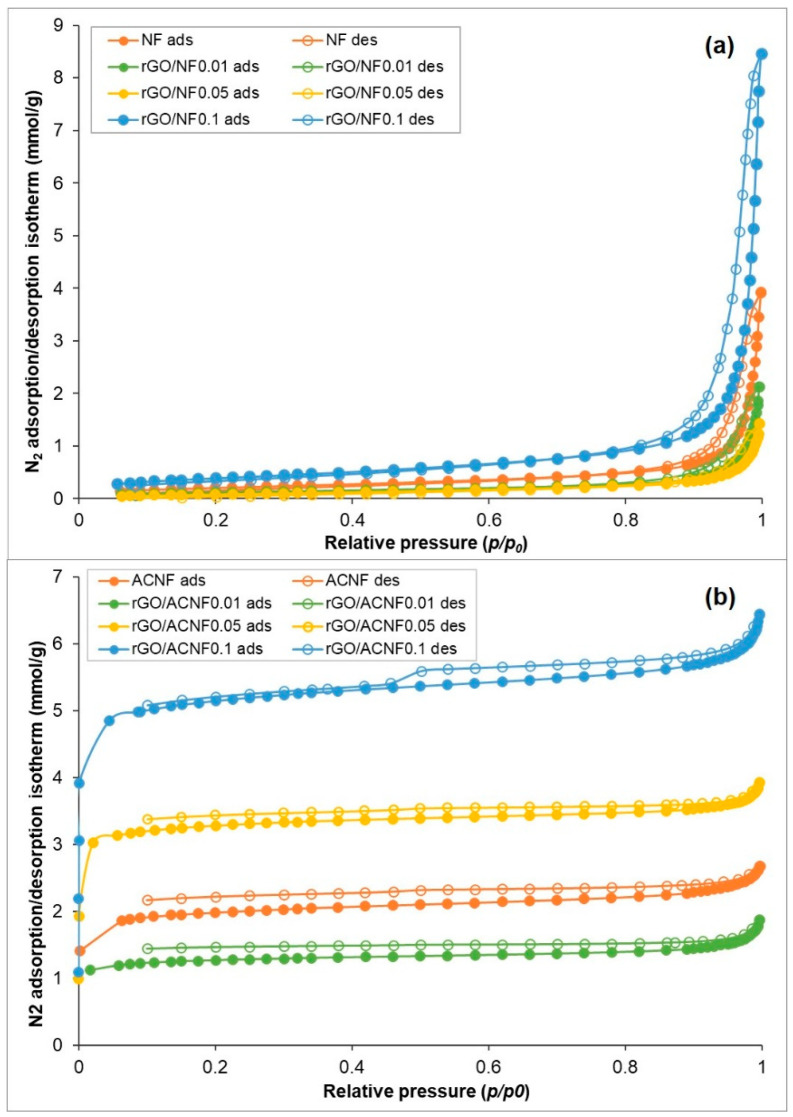
N_2_ adsorption/desorption isotherms of the materials under investigation (**a**) materials before activation and (**b**) materials after activation.

The microstructure and textural properties of nanofibers and composites before (NF and rGO/NF) and after activation (ACNF and rGO/ACNF) were studied by N_2_ adsorption/desorption analysis at −196 °C, and the plotted results are shown in Figure 2 and Table 1. Figure 2a shows the N_2_ adsorption/desorption isotherms of the NF samples before activation, showing profiles which point out type II isotherms, indicating the presence of macroporous or non-porous structures according to International Union of Pure Chemical and Applied Chemistry (IUPAC) [38,39]. This type of isotherm is believed to have single layer to multilayer adsorption, which is reflected by the shape of the isotherms in Figure 2a, i.e., linear at *p*/*p*_0_ < 0.8 and convex at *p*/*p*_0_ > 0.85–1.0. As shown in Table 2, all NF and rGO/NF materials are not microporous, i.e., *V*_micro_ = 0 cm^3^/g and the addition of rGO did not alter the textural property of the NFs, since the relevant property of rGO/NF materials does not correlate with the loading of rGO. The activation procedure was able to improve the textural property of the ACNFs. The development of meso- and micropores and increment in surface area were measured in all activated samples. The microporous properties in all samples are reflected by the Type I(b) isotherms measured by N_2_ adsorption [38,40]. As shown in Figure 2b, the sorption curves for the activated samples appear above the curves obtained in the case of non-activated materials, at least at *p*/*p*_0_ < 0.9. Besides, they show a very long plateau from *p*/*p*_0_ = 0.05–0.95, showing the typical adsorption behaviour of microporous materials. Meanwhile, at *p*/*p*_0_ > 0.95, the adsorption curve is further increased without any significant halted observed. The adsorption-desorption isotherms of ACNFs also show the presence of H3 type of hysteresis loop [38] in the *p*/*p*_0_ range of 0.4–1.0, which suggests the presence of capillary condensation in mesoporous features [41,42]. It is worth mentioning that the values of *V*_micro_/*V*_total_ confirmed that all activated samples were primarily microporous, with a relatively high proportion of *V*_micro_ to the *V*_total_. Contributions by the mesopores to the porous structure were also measured, as shown by the *V*_meso_ values.

The creation of meso-/micro-porous structures through activation at 700 °C was reflected by the significant improvement of *S*_BET_ in all samples, as shown in Table 2. *S*_BET_ of ACNFs drastically increased from 17 m^2^/g to 139 m^2^/g, which is eight times higher than the pristine NFs. For the activated composites, the highest *S*_BET_ of 373 m^2^/g was measured for rGO/ACNF0.1 and the lowest *S*_BET_ of 92 m^2^/g was determined for rGO/ACNF0.01, respectively, as rGO/ACNF0.1 exhibits the highest *S*_BET_ compared to other samples, indicating the possible high adsorption capacity. Interestingly, the effect of the amount of rGO loading was more noticeable after activation. This is could possibly be due to the catalytic effect of rGO that takes place at elevated temperatures [43]. *S*_BET_ is low in all samples before activation, showing no significant catalytic ability, but increases after being activated, indicating that rGO have good catalytic ability for developing *S*_BET_ at elevated temperatures. After activation, the relevant porous characteristics, such as *S*_BET_ and pore volumes, seem to have a good correlation with the amount of rGO loading. Typically, it is believed that adsorbent materials with high *S*_BET_ and *V*_micro_ are beneficial to adsorption of gas molecules [44].

**Figure 3:**
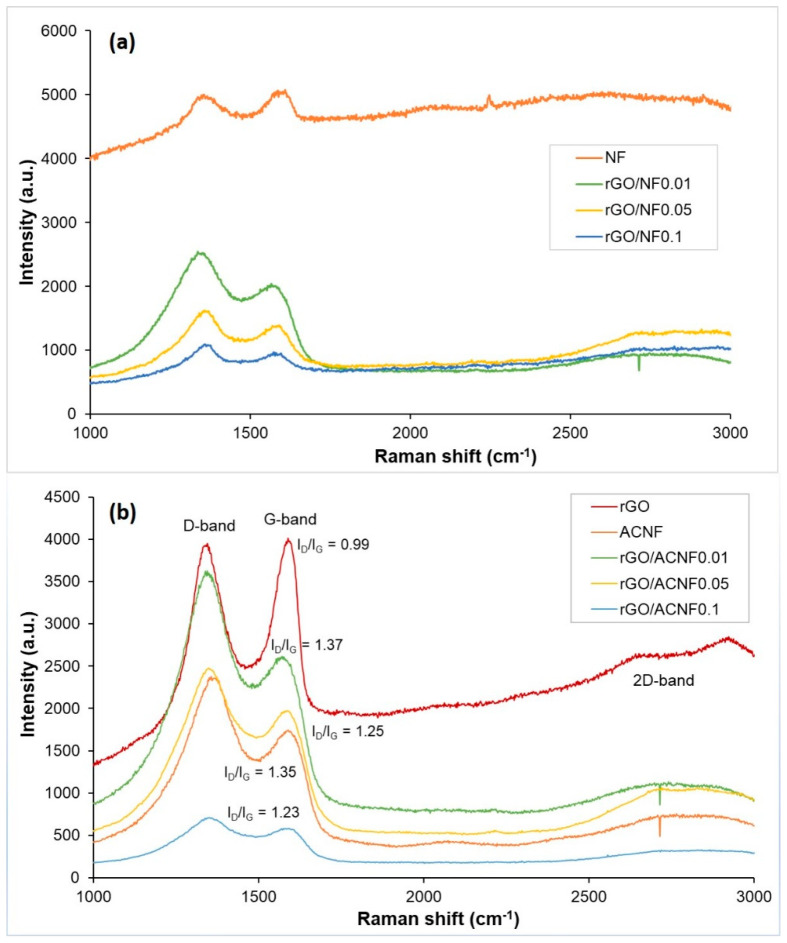
Raman spectra of rGO, ACNF and rGO/ACNF. (**a**) materials before activation and (**b**) materials after activation.

The degree of graphitization and structural features of the pristine rGO, NF and rGO/NF composites before and after activation was analyzed by Raman spectroscopy, as shown in Figure 3a,b, respectively. It can be seen that all samples under investigation exhibit two characteristic peaks at 1368 cm^−1^ and 1612 cm^−1^, which represent the D-band and G-band of graphitic materials, respectively. The presence of D-band in all samples indicates the presence of defective or disordered carbon structures [37], whilst the existence of G-band is attributed to the ordered graphitic structures with sp^2−^ hybridized carbon atoms [26]. The only significant different observed in Raman spectra before and after activation is the higher Raman intensity and more amorphous structure exhibited by the samples before activation. Reduction in intensity was observed when the samples underwent activation. In pristine rGO, both D-/G-bands have relatively strong intensities, with the appearance of weak 2D-band around 2700 cm^−1^ [45], which is typical in graphene materials. The intensity of G-band is slightly higher than that of the D-band, indicating that the self-synthesised graphene-based material from graphite demonstrated the crystalline, ordered but also possessed defect structures within the carbon lattice [46]. The non-appearance of 2D-band in rGO/ACNF composites is believed to be due to the amount of rGO composited into the structures being too small. Obviously, the intensity of D-band is higher than G-band in all ACNF-based samples, indicating that the resulting composite materials possess defective structures and are highly amorphous in nature, which is in agreement with the findings by XRD analysis (to be discussed later). 

The degree of graphitization of the materials was evaluated by calculating the intensity ratio of the D and G band (I_D_/I_G_). Obviously, rGO shows the smallest I_D_/I_G_ ratio of 0.99 compared to other electrospun materials, which represents the high degree of graphitization and ordered carbon structures in it [31,47]. In comparison with rGO, the I_D_/I_G_ of ACNF is comparatively higher, at 1.35, suggesting a less ordered carbon structure in the ACNF. As expected, the incorporation of rGO with ACNF gave the materials with I_D_/I_G_ ranging from 1.2–1.4 and this ratio decrease with the increase of the rGO concentrations (Figure 3). From the results obtained, it was demonstrated that the graphitic structure of rGO/ACNF composites were improved with the increment of rGO loading, and it was found that rGO/ACNF0.1 unveiled the smallest ratio of 1.23. Regardless of its lowest I_D_/I_G_ compared to other samples, it is believed that the carbon materials with this value are still considered to own disordered and defective structures. This is supported by a study reported by Gayathri et al. [45], as the graphene synthesized in their research still possessed a defect structure, even at very low I_D_/I_G_ of ~0.2–0.4. 

**Figure 4:**
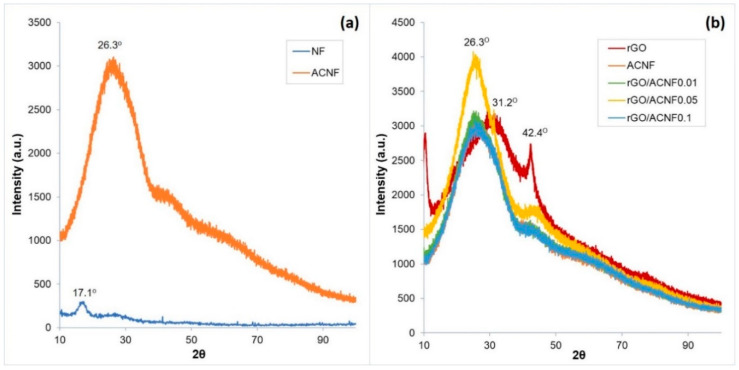
XRD spectra of (**a**) pristine NF and ACNF; and (**b**) rGO, ACNF, and rGO/ACNFs.

Figure 4a shows the comparative XRD patterns of NF and ACNF. XRD spectrum of NF shows a peak at 17.1°, which can be attributed to the diffraction feature of PAN as polymer precursor [48,49]. After activation at 700 °C, the presence of broad peak at 26.3° at high intensity in ACNF corresponds to the 002 plane of the carbon skeleton [50]. This means that carbon-based materials with ladder structures such as ACNFs have been successfully developed through the cyclization of PAN during activation. Activation has also improved the crystallinity of the ACNFs. However, the ACNFs obtained in this study still remain amorphous (absence of sharp peak) due to the destruction of the in-plane aromatic lattices [51], leaving highly defect structures in the ACNFs [2,26]. Meanwhile, Figure 4b shows the comparative XRD patterns of rGO, ACNFs, and rGO/ACNF composites, which were used to investigate the effect of rGO loading on the graphitization degree and their crystallinity. All materials under investigation, including the rGO, show one intense broad diffraction peak in a range of 17°–31° (hexagonal graphite), and another characteristic peak at 41°–43° (rhombohedral graphite) [40], with variation only in their intensities. XRD analysis of carbonaceous phase in the materials can tell the feature of the crystallographic (100) and amorphous (110) planes. As previously mentioned, the appearance of the low and broad peaks at 41°–43° represents the amorphous structure with a very low graphitization degree. This result corresponds well to the finding by Raman spectroscopy. According to a study conducted by Guo et al. [52], carbon materials with amorphous and defective structures are believed to have more potential in altering the textural properties of the ACNFs, which can improve their adsorption capacity. Therefore, the ACNFs with the defective nature developed by this study were believed to have high CO_2_ adsorption capacity.

**Figure 5:**
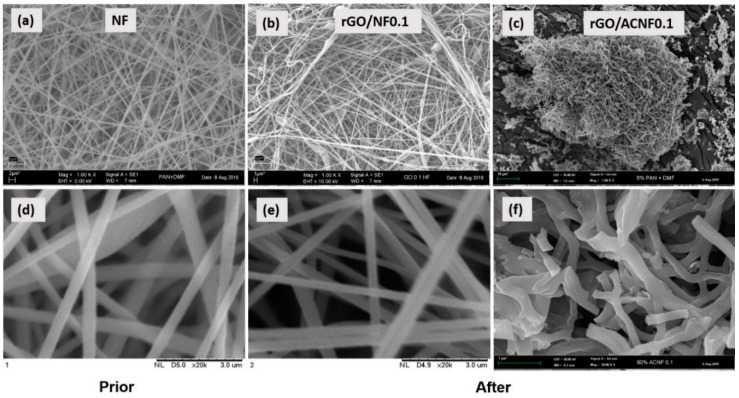
FESEM micrographs of NF, rGO/NF0.1 and rGO/ACNF0.1 composite at different magnifications (**a**–**c**) of 1000× magnification and (**d**–**f**) of 20,000× magnification.

Figure 5 shows the morphology and structure of NF, rGO/NF0.1, and rGO/ACNF0.1 composites at two different magnifications, i.e., 1000× and 20,000×. The electrospun NF and rGO/NF0.1 exhibited smooth, thread-like and interconnected fibrous structures as shown in Figure 5a,b,d,e. In Figure 5e, the incorporation of 10% of rGO (Figure 5e) shows a reduction in their average diameter from ~450 nm to ~280 nm, as compared to their pristine NF. A few droplets were detected in some regions in NF composite, as shown in Figure 5b, and this is due to the effect of rGO, because graphene-based materials must alter the conductivity as well as viscosity of the solution to be electrospun [53]. Subsequently, these conductivity changes should affect the electrostatic repulsion among jet spray from the needle tip to the collector surface during the electrospinning process, which affecting the final diameter of the fibers. A report by Huang et al. [54] stated that the solution with high conductivity should lead to production fibers by electrospinning with smaller diameter. Since it seems that the smaller the fiber diameter, the higher the S_BET_, it is expected that one could be able to control the S_BET_ by simply changing the conductivity of the polymer solution to be electrospun with the simple addition of rGO. In this study, the production of materials with high S_BET_ is one of the important issues, since it is one of the factors which contribute to the high adsorption capability. On the other hand, the change of morphology and the further reduction in fibers diameter were also observed after activation at 700 °C, as shown in Figure 5c,f. This result is in accordance with those obtained by Kim et al. [44]. The ACNFs become coarser, wrinkled, and shrunk when activated, but still maintain its fibrous morphology with some fracture identified. The shrinkage in the diameter is caused by the evolution of volatile species during the cyclization of the ACNFs, as well as the removal of moisture [55]. 

**Figure 6:**
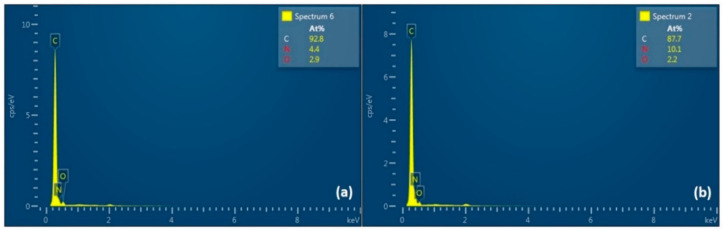
EDX spectra of (**a**) pristine ACNF and (**b**) rGO/ACNF composites.

Elemental dispersive X-ray (EDX) analysis of the pristine and composite ACNFs was shown in Figure 6. Both EDX spectra revealed similar composition of three elements, which were carbon (C), nitrogen (N), and oxygen (O), but with different percentages. An insignificant difference was detected in the elemental composition between the two samples, as they were both carbon-based materials from a similar carbon precursor. The percentage of C in pristine ACNF is 92.8%, which is higher than than of rGO/ACNF, that is, 87.7%. This was probably due to the comparatively high concentration of PAN polymer (as carbon-based precursor) used in preparing the dope solution, resulting in a high C element in the structure.

### 3.2. CO_2_ Adsorption Performance

**Figure 7:**
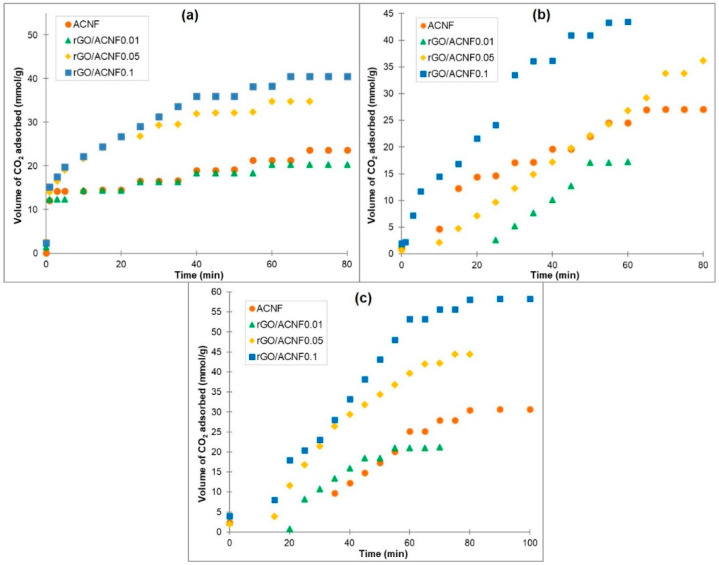
CO_2_ uptake kinetics of the pristine ACNF and composite at different adsorption pressures (**a**) 5 bar, (**b**) 10 bar, and (**c**) 15 bar at 25 °C.

**Figure 8:**
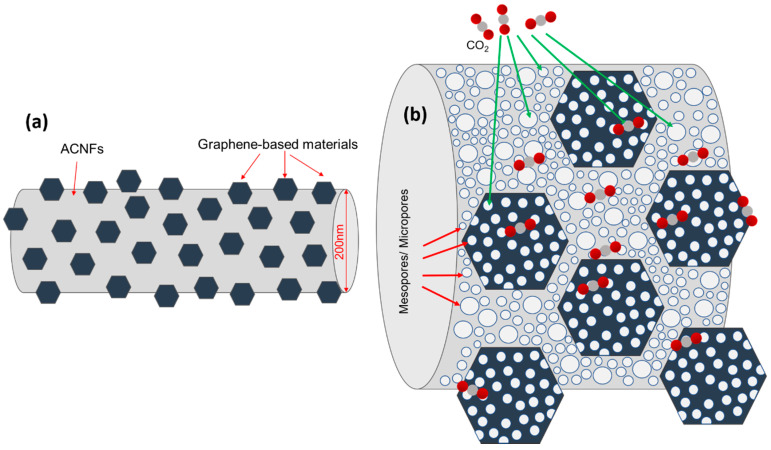
Illustration of (**a**) overview of ACNFs composite structure and (**b**) CO_2_ adsorption mechanism on the gACNFs.

The adsorption capacity as a function of time and detailed parameters of the CO_2_ uptakes of the pristine ACNF and composites are summarized in Figure 7 and Table 2, respectively. Figure 7 shows that the shape of CO_2_ adsorption curves of all materials under investigation is quite similar, with only difference in the amount adsorbed. It was found that, after the introduction of CO_2_, 95% of adsorption happened within one hour, which was followed by the slow uptake until the equilibrium. This can be explained by the increased diffusion resistance during adsorption and the reduction in the unoccupied active sites in the ACNFs. Figure 8 represents the adsorption mechanism between the CO_2_ molecules and the ACNFs. The CO_2_ molecules were adsorbed onto the available microporous and mesoporous structures in the ACNFs by van der Waals forces of attraction, forming either monolayer or multilayers, depending on the behavior of the adsorption. 

All samples exhibited similar trend at all pressures, as follows: rGO/ACNF0.01<ACNF<rGO/ACNF0.05<rGO/ACNF0.1. At 5 bar, the CO_2_ uptakes of rGO/ACNF0.1 are the highest amongst the other composites, which is almost doubled the value of the uptakes of the ACNF, i.e., 41 mmol/g vs. 24 mmol/g. With an increase in the adsorption pressure, the CO_2_ uptake increased as well, suggesting that the capture of CO_2_ by this kind of adsorbents can be described as a physisorption phenomenon [56,57]. As shown in Table 3, the CO_2_ uptakes of rGO/ACNF0.1 increases from 41 mmol/g at 5 bar to 43 and 58 mmol/g at 10 and 15 bar, respectively. Interestingly, in spite of the moderate S_BET_ obtained, the adsorption value is comparatively higher than any previously reported on CO_2_ adsorbent materials, which makes it a potentially excellent future CO_2_ adsorbent. In fact, the adsorption isotherms obtained from the various pressures and specific temperature explained the equilibrium adsorption capacity of the ACNFs. The isotherms were found to be Type I, being in line with the findings based on N_2_ isotherms, i.e., the microporous features that were induced to achieve the high adsorption of CO_2_ [58]. 

Table 3 summarized the comparison between CO_2_ uptakes by different carbon fibers-based adsorbents and their composites from previous literature with the rGO/ACNF composites produced in this study. As shown, the resultant *S*_BET_ of the rGO/ACNF0.1 is the lowest among the other reported adsorbents. Regardless, its low S_BET_ value, the volume of the CO_2_ uptakes is the highest. This excellent adsorption performance is highly believed due to its well-distributed porous structures, which is the *V*_micro_ occupied up to 68% of the *V*_total_ and another 32% was occupied by mesoporous. Both micropores and mesopores play vital role in the adsorbates-adsorbent interaction. 

### 3.3. Adsorption Kinetics

**Figure 9:**
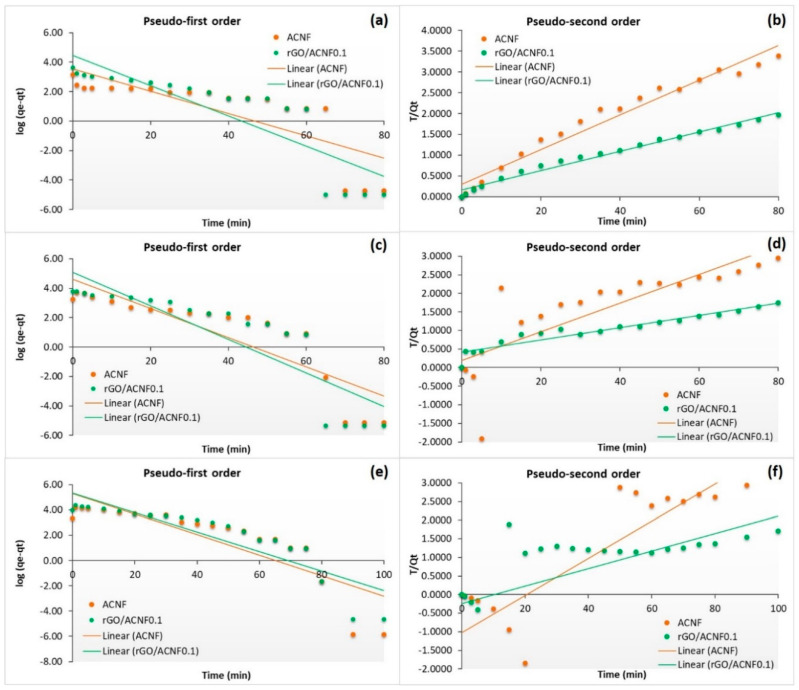
Pseudo-first order and pseudo-second order kinetic models of ACNF and rGO/ACNF0.1 composite at different pressures: (**a**,**b**) 5 bar, (**c**,**d**) 10 bar, and (**e**,**f**) 15 bar.

For the adsorption of CO_2_ by ACNF and rGO/ACNF0.1 at different pressures (5, 10, and 15 bar), Figure 9 shows the representation of experimental data and linear fittings associated with the kinetic models presented in the experimental part. In pseudo-first order model, a plot of log(*q*_e_-*q*_t_) versus *t* will generate a straight line using Equation (2). Meanwhile, in pseudo-second order model, the straight-line plot of *t*/*q*_t_ against *t* was generated using Equation (3). On the other hand, Table 4 gathers the kinetic parameters obtained from the linear regressions and the corresponding correlation coefficients (R^2^). Based on the R^2^ values collected in Table 4, the pseudo-second order model could be appropriate to describe the adsorption behavior of ACNF at 5 bar and rGO/ACNF0.1 at 5 and 10 bar, while the pseudo-first order model fitted the adsorption data best for the pristine ACNF at 10 and 15 bar and rGO/ACNF0.1 at 15 bar. The low of R^2^ values found in the samples at certain pressures shown in Table 4 represents the bad quality of linearization [63] and verifies that these kinetic models do not fit well with the experimental data of the CO_2_ adsorption depending on the specific pressures used. Therefore, it seems that, when the pressure of CO_2_ is high enough, the CO_2_ adsorption process can be better described by the pseudo-first order kinetic model, implying therefore mainly a physisorption phenomenon. On the contrary, when the pressure of CO_2_ is low enough, the adsorption phenomena is better described by chemical reaction between the free sites on the ACNF and the CO_2_ molecules, chemisorption [64]. It can be said that both ACNFs and rGO/ACNF0.1 are well-fitted pseudo-second order model at low pressure adsorption with higher R^2^ values of 0.9645 and 0.9825, respectively, compared to pseudo-first order model. In fact, lots of previous studies have mentioned that the CO_2_ adsorption onto the sorbents’ surface at higher pressure involve physisorption rather than chemisorption, which is more related to the adsorption towards the surface with higher *S*_BET_, *V*_total_, and *V*_micro_ by weak dipole interactions [65,66]. Furthermore, it also believed that this physisorption, at higher adsorption pressure, resulted in the development of multilayers of CO_2_ molecules on the heterogeneous surface of both ACNFs samples. For example, a study by Belmabkhout et al. [12] reported that the adsorption of pure CO_2_ (14.7 mmol/g) onto the sorbents’ surface at high pressure (e.g., 45 bar) and ambient temperature involved reversible physisorption. In conclusion, it can be said that these two kinetic models are found to be suitable for fitting the present kinetic data of CO_2_ adsorption at 15 bar and 25 °C, in the following order: Pseudo-first order > pseudo-second order.

## 4. Conclusions

The excellent CO_2_ adsorbent was successfully fabricated by incorporating reduced graphene oxide (rGO) into the PAN-based activated carbon nanofibers (ACNF) via the simple electrospinning and activation methods. It is noteworthy to mention that the suitable amount of rGO composited into the ACNFs is very crucial in producing rGO/ACNF composites with outstanding physicochemical properties, as well as high adsorption capacity. It worth to mention that the suitable loading of rGO is very crucial to obtain the ACNFs composites with the best properties and adsorption performance. In this study, the rGO/ACNF0.1 with 10% of rGO shows higher CO_2_ uptakes of 58 mmol/g at 15 bar and 25 °C, which almost double the uptakes value compared to ACNF with a moderate *S*_BET_ value of 373 m^2^/g. At higher pressure, the CO_2_ adsorption obeyed the pseudo-first order kinetic model, indicating the physisorption between the CO_2_ molecules and the porous network of the adsorbents. This is the leading report on PAN-rGO/ACNF composites fabricated from self-synthesized graphene-based materials, such as rGO and porous carbon materials such as ACNFs, which is an efficient way to discover low-cost carbon adsorbents for a high CO_2_ uptake.

## Figures and Tables

**Table 1:** Porous characteristics of the pristine nanofibers (NF) and reduced graphene oxide (rGO)/NFs nanocomposites before and after activation.

	Samples	*S*_BET_ (m^2^/g)	*V*_total_ (cm^3^/g)	*V*_micro_ (cm^3^/g)	*V*_meso_ (cm^3^/g)	*V*_micro_/*V*_total_ (%)
**Before Activation**	NF	17	0.14	0	0	0
	rGO/NF0.01	10	0.07	0	0	0
	rGO/NF0.05	8	0.05	0	0	0
	rGO/NF0.1	32	0.30	0	0	0
**After Activation**	Activated carbon nanofibers (ACNF)	139	0.09	0.06	0.03	67
	rGO/ACNF0.01	92	0.07	0.04	0.03	57
	rGO/ACNF0.05	233	0.14	0.09	0.05	64
	rGO/ACNF0.1	373	0.22	0.15	0.07	68

*S*_BET_ = specific surface area; *V*_total_ = total pore volume; *V*_micro_ = micropore volume; *V*_meso_ = mesopore volume.

**Table 2:** CO_2_ uptakes at different pressures at ambient temperatures.

Sample	CO_2_ Uptakes (mmol/g)		
	5 bar	10 bar	15 bar
ACNF	24	27	31
rGO/ACNF0.01	17	20	21
rGO/ACNF0.05	34	36	44
rGO/ACNF0.1	41	43	58

**Table 3:** Comparison of CO_2_ adsorption capacity on various types of carbon fibers and their composite-based adsorbents.

Materials	*S*_BET_ (m^2^/g)	*V*_micro_ (cm^3^/g)	Vol. of CO_2_ Adsorbed (mmol/g)	Temp; Pressure	Ref.
rGO/ACNF0.1	373	0.22	41	25 °C; 5 bar	This work
			43	25 °C; 10 bar	
			58	25 °C; 15 bar	
PVDF-CNFs	1065	0.61	3.1	30 °C; 1 atm	Hong et al., 2014 [58]
CNF-SnO2	434	0.20	2.6	25 °C; 1 atm	Ali et al., 2020 [54]
CNF-MIL-53	140	-	1.35	25 °C; 1 bar	Ullah et al., 2014 [59]
PAN-CMFs	966	-	2.9	25 °C; 1 bar	Ojeda-Lopez et al., 2019 [60]
Graphite NFs	567	0.27	59.2 mg/g	25 °C; 1 atm	Meng and Park, 2010 [61]
Graphite NFs	966	0.25	70.8 mg/g	25 °C; 1 atm	Yuan et al., 2016 [62]

**Table 4:** The fitted parameters of pseudo-first order and pseudo-second order of ACNF and rGO/ACNF0.1 composite.

Sample	Pressure (bar)	*q*_e,exp_ (mmol/g)	Pseudo-First Order		Pseudo-Second Order	
			*k*_1_	*R* ^2^	*k*_2_	*R* ^2^
ACNF	5	24	0.1036	0.6337	0.1397	0.9645
rGO/ACNF0.1		41	0.0865	0.7566	0.1435	0.9825
ACNF	10	27	0.1217	0.7595	0.1902	0.6563
rGO/ACNF0.1		46	0.0941	0.7667	0.0531	0.9127
ACNF	15	31	0.1114	0.7266	0.0319	0.3897
rGO/ACNF0.1		58	0.0716	0.7861	0.0697	0.2493

## Supplementary Materials

The following are available online at https://www.mdpi.com/2073-4360/12/9/2117/s1, Table S1: Dope formulation of gACNFs composite with different concentrations of reduced graphene oxide (rGO).
