# Supplementary material for: Electrospun Composites Made of Reduced Graphene Oxide and Polyacrylonitrile-Based Activated Carbon Nanofibers (rGO/ACNF) for Enhanced CO2 Adsorption
Source: Polymers (Basel). 2020 Sep 17;12(9):2117. doi: 10.3390/polym12092117 (PMC7569857; doi:10.3390/polym12092117)
Supplement: Supplementary file 1 [file polymers-12-02117-s001.pdf]

polymers

Electrospun Composites Made of Reduced Graphene Oxide and Polyacrylonitrile-Based Activated Carbon Nanofibers (rGO/ACNF) for Enhanced CO_2 Adsorption

Faten Ermala Che Othman [1], Norhaniza Yusof [1], Javier González-Benito [2,*], Xiaolei Fan [3] and Ahmad Fauzi Ismail [1]

[1] Advanced Membrane Technology Research Centre (AMTEC), School of Chemical and Energy Engineering, Faculty of Engineering, Universiti Teknologi Malaysia, 81310 Johor Bahru, Malaysia; fermala2@live.utm.my (F.E.C.O.); norhaniza@petroleum.utm.my (N.Y.); afauzi@utm.my (A.F.I.)
[2] Department of Materials Science and Engineering and Chemical Engineering, IQMAAB, Universidad Carlos III de MadridAvda. Universidad, 15, Leganés, 28911 Madrid, Spain;
[3] Department of Chemical Engineering and Analytical Science, School of Engineering, The University of Manchester, Manchester M13 9PL, UK; xiaolei.fan@manchester.ac.uk
* Correspondence: javid@ing.uc3m.es

Supplementary Information

Table S1. Dope formulation of gACNFs composite with different concentrations of reduced graphene oxide (rGO).

Sample	Concentration of rGO (% relative to PAN wt.)	PAN (% reladtive to total wt.)	DMF (% relative to total wt.)
**NF**	0	8	92
**rGO/NF0.01**	1	8	92
**rGO/NF0.05**	5	8	92
**rGO/NF0.1**	10	8	92

© 2020 by the authors. Licensee MDPI, Basel, Switzerland. This article is an open access article distributed under the terms and conditions of the Creative Commons Attribution (CC BY) license (http://creativecommons.org/licenses/by/4.0/).
